# Schizophrenia, the gut microbiota, and new opportunities from optogenetic manipulations of the gut-brain axis

**DOI:** 10.1186/s12993-021-00180-2

**Published:** 2021-06-22

**Authors:** Enrico Patrono, Jan Svoboda, Aleš Stuchlík

**Affiliations:** Institute of Physiology of the Czech Academy of Sciences, Videnska, 1830, Prague, 142 20 Czech Republic

**Keywords:** Schizophrenia, Gut microbiota, NMDA hypoactivity, NMDARs/GABA interaction, Probiotic dietaries, Fecal microbiota transplantation, Gut optogenetics

## Abstract

Schizophrenia research arose in the twentieth century and is currently rapidly developing, focusing on many parallel research pathways and evaluating various concepts of disease etiology. Today, we have relatively good knowledge about the generation of positive and negative symptoms in patients with schizophrenia. However, the neural basis and pathophysiology of schizophrenia, especially cognitive symptoms, are still poorly understood. Finding new methods to uncover the physiological basis of the mental inabilities related to schizophrenia is an urgent task for modern neuroscience because of the lack of specific therapies for cognitive deficits in the disease. Researchers have begun investigating functional crosstalk between NMDARs and GABAergic neurons associated with schizophrenia at different resolutions. In another direction, the gut microbiota is getting increasing interest from neuroscientists. Recent findings have highlighted the role of a gut-brain axis, with the gut microbiota playing a crucial role in several psychopathologies, including schizophrenia and autism.

There have also been investigations into potential therapies aimed at normalizing altered microbiota signaling to the enteric nervous system (ENS) and the central nervous system (CNS). Probiotics diets and fecal microbiota transplantation (FMT) are currently the most common therapies. Interestingly, in rodent models of binge feeding, optogenetic applications have been shown to affect gut colony sensitivity, thus increasing colonic transit. Here, we review recent findings on the gut microbiota–schizophrenia relationship using in vivo optogenetics. Moreover, we evaluate if manipulating actors in either the brain or the gut might improve potential treatment research. Such research and techniques will increase our knowledge of how the gut microbiota can manipulate GABA production, and therefore accompany changes in CNS GABAergic activity.

## Background

In 1911, Eugene Bleuler published his monograph on schizophrenia (SCZ) [1], driving the neuroscientific exploration of SCZ toward a psychological rather than a neural basis, and devoting more space for symptoms than for causes [2]. Further, Bleuler defined several “schizophrenias”, reflecting the ability of SCZ to manifest with several clinical conditions.

Over the last century, the neuroscience of SCZ has developed on various research pathways in parallel, evaluating other ideas for the etiology of the disease, from mental symptoms to a theory of mind and social cognition [3]. Nowadays, we know the brain substrates of both positive and negative symptoms in patients with SCZ. However, modern neuroscience needs to investigate the neural basis of cognitive deficits in more detail, as these are strongly viewed today as a primary and predicting long-term outcome of the disease [4–6]. Recently, several authors have elucidated a functional relationship between N-methyl-D-aspartate subtypes of glutamate receptors (NMDARs) and gamma-aminobutyric acid (GABA) at different levels. The “NMDAR hypofunction hypothesis” is particularly fascinating, based on evidence that the use of NMDAR antagonists induces SCZ-like positive, negative, and cognitive symptoms in healthy subjects and may exacerbate these symptoms in SCZ patients [7]. Several animal models have used phencyclidine (PCP) and ketamine to induce SCZ-like symptoms, including psychosis and cognitive dysfunction in rodents [8, 9]. From post-mortem studies to genetics, a wide range of findings has revealed a significant role in GABAergic transmission [10–14]. The potential NMDARs/GABA relationship provides insights into how GABAergic inhibitory dysfunction may mediate alterations of the function of NMDARs and vice versa. In turn, this can lead to maladaptive interactions between brain regions, a crucial hallmark of SCZ [15].

The etiology of SCZ remains unclear, yet it is clear that both genetics and environmental factors play critical roles in developing this condition. Several findings have revealed associations between flawed immune system regulation and SCZ [16, 17]. For example, many SCZ patients suffer from gastrointestinal (GI) tract problems [18, 19]. Significantly, an increase of stress-mediators, pro-inflammatory interleukin cytokines (Interleukin-1, Interleukin-6, and Interleukin-β) has been found in the gut of SCZ patients [20, 21]. This increase may represent a 2-way brain-gut communication that could affect brain cognition in SCZ through an altered gut immune system. Finally, it has been found that altered gut microbiota plays a role in SCZ-like states in rats after sub-chronic PCP administration [22], while ampicillin treatment recovered the cognitive functions. Interestingly, the penicillin group of antibiotics has direct pharmacological actions on GABA-mediated neurotransmission [23, 24]. These results contribute to increasing evidence of a gut-brain axis underlying a potential etiology of psychiatric diseases, especially SCZ.

The gut microbiota has lately been receiving much interest from neuroscientists. Recent findings highlighting the role of a gut-brain axis have shown that the gut microbiota plays a crucial role in many psychopathologies. For instance, depression and anxiety [25]; addiction [26]; eating disorders [27]; neurodegenerative disorders such as Alzheimer’s disease (AD) [28]; psychiatric disorders such as SCZ and autism [22, 29–31] can involve changes in the gut-brain axis. In humans, the gut microbiota has many more bacteria and other organisms than other body areas [32]. Besides the high amount of bacteria, they include fungi, viruses, protozoa, and archaea, and the diversity of the microbiota varies from person to person, depending on environmental factors. The gut microbiota starts to take shape from birth and during the first 1–2 years. After this period, the child’s gut microbiota diversity resembles that of adults, and yet, environmental factors may still perturb the gut microbiota balance [33]. Environmental factors such as location, surgery, smoking, depression, and living arrangements influence “microbiotic shaping.” Feeding may also affect specific bacterial groups in the infant’s gut microbiota. For example, *Bifidobacterium longum* uses oligosaccharides in mothers’ milk to compete with other bacteria such as *E. coli* and *Clostridium perfringens* [34]. An altered gut microbiota—dysbiosis—drives many disease conditions ranging from chronic GI distress to neurodevelopmental and neuropsychiatric disorders [35]. Depending on the type and the stage of disease, microbiome modulators such as prebiotics and probiotics aim to correct the dysbiosis, re-establishing effective communication between the host and the targeted microbiota.

This review will first overview the latest findings on SCZ, highlighting recent significant hypotheses and the actors investigated. We will describe the hypothesized hypoactivity of NMDARs and the role of a GABAergic dysfunction and discuss how these two players interact with each other. Then, we will discuss recent studies related to the gut microbiota and interactions with the CNS, forming the so-called gut-brain axis, and will look extensively at research that raised attention on the gut-brain axis’'s role in psychiatric disorders such as SCZ. Furthermore, we will discuss technical applications for manipulating the gut microbiota and the ENS between the gut-brain axis with probiotics dietaries and the fecal microbiota transplant (FMT) technique. We will also discuss new techniques, including optogenetics on the gut microbiota, which investigates the role of the gut microbiota in SCZ.

Interestingly, it is possible to study the gut microbiota-SCZ relationship in detail using in vivo optogenetics, manipulating either the brain or the gut actors. Indeed, to our knowledge, no study to date has systematically explored the hypotheses of an optogenetic gut manipulation to reduce central GABAergic activity, which in turn would drive cognitive deficits found in SCZ. Finally, this review will help address several questions regarding the relationship between the gut microbiota and SCZ and present new therapeutic possibilities to treat psychiatric illnesses by acting on the gut microbiota.

## Schizophrenia

The definition from the Diagnostic and Statistic Manual of Mental Disorders, 5th edition (DSM-5) states that SCZ is characterized by delusions, hallucinations, disorganized speech and behavior, and other symptoms that cause social or occupational dysfunction [36]. For a diagnosis, symptoms must have been present for 6 months and include at least 1 month of active symptoms. Causes are still mostly unknown, and no society or culture anywhere in the world is free of SCZ [37]. With an incidence of 7/10,000 people, i.e., approaching 0.7% of the population, SCZ is a severe public health problem [38]. The DSM-5 lists a wide range of associated symptoms: positive (hallucinations, delusions, disorganized thoughts, and speech); negative (anhedonia, voice flattening, struggling with personal daily life care); and cognitive impairments (moderate to severe across several domains, including attention, working memory, learning, and executive functions). Researchers have shown that specific factors seem to increase the risk of developing or triggering the disorder, including a combination of genetics, brain chemistry, and the environment [39–42]. Among other determinant conditions, prenatal and postnatal risk factors are the most investigated, showing that environmental vulnerabilities are crucial keys driving SCZ [43]. Our poor understanding of the physiological features of SCZ is reflected in the drug development of antipsychotics as therapeutic strategies, reducing mainly the positive symptoms rather than the negative ones or the cognitive impairments [44, 45].

### Relevant causes inducing SCZ

The wide range of risk factors contributing altogether to the onset and the development of the disorder is the peculiarity of SCZ, and researchers have constantly screened it. However, a complementary and synthesized interpretation of the combination of the several risk factors driving SCZ is still missing. Among the relevant causes inducing SCZ, prenatal and postnatal environmental vulnerabilities and the immunity system reactions are the most investigated. For example, maternal immune activation (MIA), which refers to a maternal immune system triggered by infectious or infectious‐like stimuli, has been recently considered as a “neurodevelopmental primer" acting during specific gestational timings and increasing the risk for SCZ in offspring [46–49]. Notably, a set of studies inferred that a cascade of cytokines and immunologic alterations are transmitted to the fetus, resulting in adverse phenotypes, most notably in the central nervous system [50, for review]. In addition, these studies implied that maternal respiratory infections, influenza, *Toxoplasma gondii* infections, and others are possible biomarkers of MIA, and the inflammatory mediators released following all types of infections may be fundamentally involved in the etiology of SCZ in offspring [46]. The viral mimic polyinosinic–polycytidylic acid [Poly (I:C)] is used to create an animal model of MIA, and it is recently emerging as a highly potent preclinical research tool in the quest for both symptomatic and preventive treatment approaches [50]. Poly (I:C) is a sodium salt-based immunostimulant structurally similar to a double-stranded RNA virus, interacting with toll-like receptors and therefore activating the immune system. However, if this approach offers advantages in inducing a temporally controlled infection in pregnant rodents, it falls in reproducing the whole spectrum of possible immune responses after infections.

Regarding the postnatal risk factors, many studies focused on early life stress, such as malnutrition and maternal separation [40, 51]. Remarkably, animal models of maternal deprivation have been used to address several behavioral and neurochemical changes in the offspring’s brains. For example, increases in serotonin in the hippocampus (HPC), striatum, and prefrontal cortex (PFC) can result in dampened functions of the hypothalamic–pituitary–adrenal (HPA) axis in periadolescent rats [52, 53], which in turn is linked to depression, memory dysfunctions, and SCZ. Moreover, NMDAR subunits expression is altered after maternal deprivation, impairing cognitive abilities associated with SCZ later in adult life [54].

Namely, an NMDAR antagonist (memantine) administration altered social cognition in adult rats who underwent early maternal deprivation [55]. However, a further study using chronic administration of another NMDAR antagonist (MK-801) in juvenile rats showed impaired working memory but not changes in the expression levels of NMDAR subunits, supporting a face validity rather than construct validity of the model, and therefore arguing that only drug treatment is not sufficient for an animal model of early life onset of SCZ [56]. Finally, another study confirmed that administration of MK-801 in infancy and social isolation in childhood are two independent factors on the neurodevelopmental defects [57].

Collectively, these reports indicate that pre and postnatal risk factors have broad effects on neurotransmitter and neuroendocrine systems, which is likely key for the expression of altered cognitive function associated with SCZ later in life.

### The role of N-methyl-D-aspartate hypoactivity, GABAergic dysfunction, and their interaction

In the past few decades, different hypotheses have been raised to explain the neurophysiological etiology of SCZ. Of particular interest is the “NMDAR hypofunctioning hypotheses”, based on evidence reporting that NMDAR antagonists can induce positive and negative symptoms and cognitive impairments resembling SCZ in healthy subjects and exacerbate the psychotic symptoms in patients [7]. NMDARs are tetrameric ionotropic receptors, composed of two GluN1 subunits (containing the glycine/d-serine binding site) and two GluN2 subunits (containing the glutamate/NMDA binding site). They are crucial in glutamatergic neurotransmission and synaptic plasticity, thus modulating cognition, memory, and prefrontal executive functions [58–60]. For instance, a double-blind, between-subjects design evaluated 32 healthy control participants matched to 32 psychotic patients with SCZ [61]. The task required participants to sample individual amounts of sensory information to infer correct decisions or provide explicit probability estimates for the presented sensory information. Results showed that healthy participants receiving dextromethorphan, an NMDAR antagonist, displayed a “jumping-to-conclusions” bias, abnormally increased probability estimates, and overweighting of sensory information. These effects were similar to those from patients with SCZ performing identical versions of the task. These results provided novel neuropharmacological evidence linking reduced glutamatergic neurotransmission to impaired information sampling and disrupting probabilistic reasoning, namely to overweighting sensory evidence, in patients with SCZ. Another human study aimed at testing the effects of ketamine, another NMDAR antagonist, on resting-state activity using magnetoencephalography (MEG) in healthy volunteers [62]. The researchers reported elevated gamma activity in the thalamus and hippocampus, and the frontal and temporal cortex regions. Conversely, reductions in beta activity were localized to the cerebellum, the anterior cingulate, and the temporal and visual cortex. These findings highlight the potential contribution of thalamus-cortical connectivity patterns in ketamine-induced neuronal dysregulation, which may be relevant for understanding SCZ as a disorder of the disinhibition of neural circuits induced by NMDAR hypofunctioning.

In the recent past, efforts have been made to develop animal models representing SCZ induced by NMDAR hypofunctioning using drug treatments with competitive and non-competitive NMDAR antagonists, such as PCP and MK-801. The PCP model can mimic SCZ-like behavior, modeling positive, negative, and cognitive impairments similar to those in humans. For this reason, the PCP model has been often used as a potent tool to test new pharmacological candidates against the disease. The model works by injecting rodents with PCP systemically twice a day, for at least a week, to have a chronic manifestation of the SCZ features. Afterward, a washout period of seven days is needed, after which the subjects exhibit SCZ-like behaviors, such as hyperlocomotion, learning and memory impairments, and disrupted executive functions [22]. Researchers use animal models of NMDAR hypofunction to clarify the neurobiology of SCZ and test new drugs for treating its symptoms [63–66]. Svoboda and colleagues tested the ability of rats to navigate flexibly on a rotating arena, avoiding a non-marked sector, by using injections of MK-801. Results showed that navigational reversal learning was still preserved in those rats injected with MK-801. However, once the task required the rats to perform a “set-shift” and navigate the rotating arena avoiding the rotating non-marked sector, the animals could not complete the task [65]. Another electrophysiological study investigating hippocampal neurons’ resting-state activity after MK-801 injections observed that MK-801 altered the temporal coordination, but not the rate, of neuronal firing. Enhanced firing coactivation has been hypothesized to be part of a disorganized discharge at a “neuronal ensembles” level, possibly leading to disorganization in information processing [63]. In line with this hypothesis, a study employing the analysis of immediate-early-gene (IEG) expression showed that the same dose of MK-801 impairing the spatial coordination of rats on the rotating arena also depletes the contextual specificity of IEG expression in hippocampal CA1 ensembles [64]. IEG expression is critical for the maintenance of synaptic plasticity and memory consolidation. It is triggered in neuronal nuclei in a context-specific manner after behavioral exploration and is used to map activity in neuronal populations. These investigations altogether demonstrate both clinically and pre-clinically that an NMDAR hypofunctioning can induce SCZ-like cognitive malfunctions such as inflexibility, poor decision making, and disorganized reasoning. Moreover, these findings may drive clinical and preclinical researchers to better understand the SCZ etiology from behavioral, pharmacological, electrophysiological, and genetic points of view.

Numerous recent findings have revealed a significant role for GABAergic transmission [15, 67, 68], considering it to be an SCZ endophenotype. Post-mortem SCZ human brains show GABA-related deficits, particularly the involvement of GABAergic neurons with the calcium-binding protein parvalbumin (PV  +) [10–12]. Moreover, recent findings have found that PFC-PV  +  neurons of SCZ subjects have lower levels of GABA-synthesizing enzyme, GAD67 [13, 14], and compromised efficiency of the perineuronal nets (PNNs) that regulate synaptic functions [12, 69]. Neurochemically, PV  +  neurons have a crucial role in regulating the activity of pyramidal neurons (PNs) by exerting robust inhibitory control [70], allowing for a high level of feedforward and feedback inhibition that serves several essential functions [71]. Moreover, PV  +  cells are keystones in the generation of gamma oscillations (30–80 Hz) [72], which are an oscillation range linked to cognition and information processing across species [73]. Optogenetic activation or silencing of PV  +  cells in different brain areas induces altered gamma oscillations, which has resulted in improvements or worsening of cognitive abilities, respectively [74, 75]. Therefore, inhibition/excitation levels have to be in an appropriate balance for healthy cognitive abilities, and PV  +  interneurons help maintain this proper balance. In line with this, it has been argued that an altered balance of the excitatory/inhibitory synaptic transmission may occur in SCZ subjects [76]. Several studies have pointed out the functional relationship between NMDARs and GABAergic neurons at different levels, from molecular to system levels. This finding demonstrates that inhibitory dysfunctions mediated by NMDARs may lead to maladaptive interactions between brain areas associated with the crucial clinical features of SCZ [15, 58, 59, 76, 77]. For example, systemic injections of MK-801 affected the spontaneous firing of putative PV  +  GABA interneurons and PNs in freely moving rats, showing a decreased firing rate of PV + GABAergic interneurons and a delayed increased firing rate of PNs, thus resulting in a paradoxical state of excitation caused by disinhibition [58].

Moreover, an elegant theoretical model called PING (Pyramidal Interneuron Network Gamma) attempts to explain the complicated relationship between GLUergic, NMDARs, and GABAergic neurons (mainly PV  +  basket cells). This model hypothesizes that the synchronization of neural activity governs the NMDAR/GABAergic neuron relationship at a gamma-band frequency, which is essential for cognitive performance [59]. The authors theorized that an unbalanced NMDAR/PV  +  ratio would result in gamma-band desynchronization, leading to SCZ-like deficits. Using this model, very recent studies have investigated the ability of optogenetic manipulation to re-establish an NMDAR/PV  +  balance as a potential therapeutic strategy to treat SCZ-like cognitive impairments [74, 78, 79]. These studies have shown that the optogenetic enhancement of PV  +  GABAergic inhibitory activity has a beneficial role on mice and rats’ attentional and executive abilities, overcoming the NMDAR hypofunction induced by NMDAR antagonists. Furthermore, our lab has conducted pilot studies investigating the role of the optogenetic activation of PV  +  GABAergic interneurons in an animal model of SCZ-like cognitive inflexibility, a central executive functional deficit in the SCZ pathological framework. Preliminary results confirm the therapeutic ability of a GABAergic activity enhancement of PV  +  interneurons to overcome NMDAR disinhibition.

## Gut microbiota

Over the past few years, the neuroscience field has started to pay more attention to how the central nervous system (CNS) connects with other physiological systems such as the enteric nervous system (ENS) and the neuroendocrine system (NES) [80]. Some have reviewed papers taken advantage of suggestive preclinical studies enlightening the pivotal role of the gut microbiota in a wide range of CNS pathologies, from mood disorders to Alzheimer's disease, from addiction to SCZ [19, 27, 81]. Here, we evaluate the current research lines investigating interconnections between the gut microbiota and SCZ, especially concerning how an altered microbiota may cause cognitive alterations inducing schizophrenia-like symptoms.

### Relationships between the gut microbiota and gut-brain axis

The bi-directional relationships of the gut microbiota and brain functions are under the control of a broad family of gut wall cells able to transduce environmental conditions (food intake, stress response) into endogenous signals via the vagal afferents and the HPA axis [82]. In turn, back transmission to the brain happens through multiple afferent pathways, from the endocrine (microbial signaling molecules and cytokines, mainly), to the neurocrine (vagus nerve and spine afferents) [83]. If acute alterations are present in this interoceptive feedback (gastrointestinal (GI) infections), they might result in transient functional brain changes [82, 83]. However, chronic alterations of the gut microbiota (high-fat diets and long-term stress) are associated with neuroplastic changes [19, 84].

### The gut microbiota and schizophrenia

Recent studies on correlations between gut dysbiosis and psychiatric diseases have noted a strong connection between SCZ and altered gut microbiota [81, 85, 86]. A central concept arising from several reviewed studies investigating the gut microbiota-SCZ relationship is that the gut microbiota is a crucial factor in the early life development and neural maturation, including immune and endocrine systems [87–90]. These physio-behavioral processes are frequently impaired in SCZ patients. For example, a prenatal microbial infection can result in 10-to-20 times increased risk of developing SCZ [91]. Moreover, it has been seen that early-life stress may produce long-lasting changes in gut microbiota, contributing to the development of abnormal neuronal and endocrine function and behavior which could play a pivotal role in the etiology of psychiatric illness [92]. A recent study revealed that treated and non-treated SCZ patients had a decreased microbiome α-diversity index compared with healthy controls. Further, germ-free mice receiving SCZ microbiome had altered glutamate/glutamine and GABA levels in HPC, displaying SCZ-relevant behaviors similar to other mouse models of SCZ involving glutamatergic hypofunction [85]. Furthermore, based on the assumption that the inflammatory response to infection has a role in the blood-gut and blood–brain communication causing psychiatric illness, it has been shown that combined *Toxoplasma Gondii*—a neurotropic protozoan parasite—and NMDAR antibody seropositivity in SCZ resulted in higher degrees of cognitive impairment as measured by tests of delayed memory [93]. Another study on humans examined the immune-inflammatory response to five different gram-negative bacteria in SCZ subjects, correlating significantly with poor performances at several cognitive tests usually used to evaluate cognitive impairments associated with SCZ [94]. A reduced gut-immunity response is connected with a reduced organization of the gut microbiota. Thus leading to psychiatric disturbs. A study using 16S rRNA sequencing found that SCZ patients had significantly reduced gut microbiota richness compared with those of the healthy controls and a distinguished gut microbiota composition among the patients with SCZ and the healthy controls. Moreover, glutamate synthase was more active in the guts of SCZ patients than in healthy controls, and high glutamate synthase activity was associated with altered gut microbiota [95]. On this line, Shen et al. [96] analyzed whether gut microbiota can be used as a biomarker to assist in diagnosing schizophrenia. After 16S rRNA sequencing, fecal microbiota analysis, and phylogenetic investigations, they found specific microbiota biomarkers for SCZ. Moreover, the composition of microbial communities present in blood across many humans diagnosed with SCZ, bipolar disorder, and sporadic amyotrophic lateral sclerosis was analyzed, and increased microbial diversity in SCZ patients has been observed compared to other disorders [97]. The authors suggested that the increased α-diversity index compared with the other groups may be due to specific phyla characteristics to SCZ. Further investigations showed that gut flora diversity between individuals (β-diversity) has increased in SCZ patients compared to the other groups, suggesting that a single phylum or microbial profile is unlikely to cause the disease-specific increase in diversity. Moreover, a recent case study found that *Clostridium* spp. increased after electroconvulsive therapy [98]. Another study investigating ultra-risk subjects used fecal samples collection and magnetic resonance spectroscopy to correlate increased Clostridiales, Lactobacillales, Bacteroidales orders, and membrane dysfunction in the brain, supporting the “membrane hypothesis” of SCZ [99]. Moreover, SCZ patients treated with risperidone, a second-generation atypical antipsychotic, for 24 weeks had significantly lower numbers of fecal *Bifidobacterium* spp., *E. coli*, *Lactobacillus* spp. compared with healthy controls, suggesting that risperidone treatment causes significant changes in certain fecal bacteria, which are likely associated with antipsychotic medication-induced metabolic changes [100]. Furthermore, another study on humans with the first episode of psychosis found a high number of *Lactobacillus* group bacteria, supporting the idea of benefit coming from the gut microbiota modulation as a treatment therapy in SCZ [101]. In this regard, Okubo et al. [102] evaluated the effect of consuming the probiotic *Bifidobacterium* breve A-1 on anxiety and depressive symptoms in patients with schizophrenia and explored its effect on immune products such as cytokines and chemokines. Results showed that four weeks of treatment increased the scores in tests measuring anxiety/depression in SCZ patients, suggesting a potential therapeutic role of probiotics in SCZ. Other studies showed that supplementation of potato starch [103] and Vitamin D [104] ameliorated the SCZ-like anxiety/depression in patients treated with atypical antipsychotics, suggesting that dietary supplements may be beneficial in restoring gut dysbiosis in SCZ.

Other examples of FMT investigations from SCZ patients into antibiotic-treated mice caused behavioral abnormalities such as psychomotor hyperactivity, impaired learning and memory in the recipient animals [105]. Interestingly, the authors also found elevation of the kynurenine–kynurenic acid pathway of tryptophan degradation in both periphery and brain and increased basal extracellular dopamine in PFC serotonin in HPC. These findings suggest that the abnormalities in the composition of gut microbiota contribute to the pathogenesis of SCZ partially through the manipulation of tryptophan–kynurenine metabolism. For a better lookup of the abovementioned studies, a table has been created to summarize the main results of the most recent research focused on the connection between the gut microbiota and SCZ (Table 1).

**Table 1:** Evidence for a role of gut microbiota in SCZ from human and animal studies

References	Subject type	Investigated actors	Methods	Results
Pyndt Jørgensen et al. [22]	Rats	Cognitive abilities (memory performances)	Sub-chronic PCP model to induce SCZ-like behaviors	SubPCP model impaired NORT
				Increased locomotor sensitivity up to 6 weeks after washout
			NORT	
			16S rRNA gene MiSeq-based high throughput sequencing	
		Locomotor activity		Gut microbiota profiles correlated to SCZ-like memory performance
				Administration of ampicillin (restoring gut microbiota) abolished the subPCP-induced memory deficit
		Gut microbiota		
Nguyen et al. [30]	Human	α-diversity	Stool sampling	Phylum level
			16S rRNA amplicon extraction protocol	↓ Proteobacteria in SCZ vs HC
			Illumina primers to target the V4 region of the 16S ribosomal RNA gene	
				Genus level
				↑*Anaerococcus* in SCZ
				↓*Haemophilus*, *Sutterella*, and *Clostridium* in SCZ
		β-diversity		
				Within SCZ
				*Ruminococcaceae* correlated with low negative symptoms
Zheng et al. [85]	Human, mice	Gut microbiota of SCZ and HC	16S rRNA gene sequencing	↓ Microbiome α-diversity index in SCZ
				↓ Disturbances of gut microbiota composition
				*Veillonellaceae* and *Lachnospiraceae* associated with SCZ severity
			Human-to-mice Gut microbiota transplant	
		SCZ-relevant behavioral phenotypes in GF mice		
			Whole-genome shotgun sequencing of cecum	GF mice receiving SCZ microbiome
				HPC ↓glutamate and ↑glutamine and GABA
			Non-targeted metabolomics analysis	
		SCZ vs HC mice microbiota analysis		
				SCZ-relevant behaviors similar to those with GLU hypofunction
Severance et al. [90]	Human	Plasma biomarkers for general inflammation and gut microbiota derived inflammation: hs-CRP, LBP, sCD14	409 SCZ individuals	Multivariate regression models
				GI and endocrine conditions was additive for LBP, with associations only when both conditions were present compared to when were absent
			Multivariate and univariate regression models	hs-CRP strongly associated with primarily endocrine conditions
				Univariate comparisons
				*S. cerevisiae* IgG levels were elevated only in GI problematic persons
		IgG antibodies to *S. Cerevisiae*, bovine milk casein, and wheat gluten		
Babulas et al. [91]	Human	Maternal G/R infections prenatal exposure and SCZ relationship in offspring	7794 offspring reported maternal G/R infections from obstetric records	Exposure to G/R infections during the periconceptional period is associated with increased SCZ risk, with adjustment for maternal race, education, age, and mental illness
			Diagnosed 71 cases of SCZ and SCZ spectrum disorders	
Dunphy-Doherty et al. [92]	Rats	Social isolation/altered gut microbiota correlation	Behavioral testing: OF/NORT, EPM, CFR, restraint	SI rats showed a few signs of anxiety phenotype (↑ locomotion, ↓ defecation, ↓ CFR). No differences in NORT and EPM
			ELISA	No changes in corticosterone after stress
		HPC neurogenesis and brain cytokine levels	16S rRNA gene MiSeq-based high throughput sequencing	↓ BrdU/NeuN in dentate gyrus
		Post-mortem caecal microbiota composition	Cytokine and mTOR analysis	↓ Il-6 and IL-10 in HPC
Kannan et al. [93]	Human, mice	*T. gondii*-induced infection in mice and human cohorts	Mice received 2 *T. gondii* strains, or NaCl (HC) i.p. injections	In mice
			Serum collection from blood tail 20 weeks post infection	*T. gondii* infection produced sustained, strain-specific, anti-NMDAR immune responses
			Enzyme-linked immunosorbent assays	In humans (USA and Germany cohorts)
		IgG class antibodies to the NMDAR		↑ NMDAR IgG levels in *T. gondii*-seropositive SCZ vs HC
			Serum IgG antibodies (*T. gondii*, NMDAR subunit)	
			Humans: 2 cohorts (USA, Germany) with	
		*T. gondii* and NMDAR antibody seropositivity		= NMDAR IgG levels in medicated *T. gondii*-seropositive SCZ vs HC
			Blood sampling	
			Subject selection with RBANS	
Maes et al. [94]	Human	Plasma IgA/IgM against 5 g-negative bacteria	Recruitment (80 SCZ, 38 HC)	IgA/IgM values
				IgA values associated with the SCZ Deficit Phenotype
			SDS screening (40 out of 80 SCZ)	No associations between IgM and the 5 Gram-negative bacteria
		IgM MDA and azelaic acid		
			Assessments (MINI, PANSS, BPRS)	Low IgM to MDA and azelaic acid in SCZ deficit phenotype
			Immunoassays for	
			IgA and IgM against 5 g-negative bacteria	
		SCZ deficit phenotype	IgM-mediated autoimmune responses directed against MDA and azelaic acid	
Xu et al. [95]	Human	GMEs	Recruitment (84 SCZ + HC)	MWAS found 19 different taxonomies in SCZ vs HC; and 12 were increased in SCZ
			Stool sampling and analysis	
				↑ MD index in SCZ vs HC
			MEs analysis	
				↑ MEs diversity in SCZ, + correlation with the MD index
		Gut microbiota taxonomies	16S rRNA gene MiSeq-based high throughput sequencing	
				↑ GOGAT in the SCZ guts
			MWAS	
				ROC analysis showed that MD index, IgA and GOGAT reached AUC 0.86 → potential gut markers of SCZ
		MD index		
			Correlation and regression analysis	
			MD analysis	
			ROC analysis	
		GOGAT		
			ELISA	
Shen et al. [96]	Human	SCZ and HC gut microbiota	16S rRNA gene MiSeq-based high throughput sequencing	Similar α-diversity SCZ vs HC
			ROC analysis	β-diversity altered → increased phylum
				Proteobacteria, Fusobacteria, Firmicutes, Bacteroidetes
		α-diversity		
			PICRUSt analysis	β-diversity altered → decreased phylum
				Mainly Firmicutes (Clostridia, Streptococcus, etc.)
		β-diversity		
Olde Loohuis et al. [97]	Human	Microbial communities composition in the blood	Recruitment (192 with SCZ, ALS, BPD) + HC	↑ Microbial diversity in SCZ vs ALS, BPD, HC
			High-quality unmapped RNA sequencing reads	
				↑ Microbial diversity is inversely correlated with estimated abundance of antigenic CD8 + T cells in HC
Kanayama et al. [98]	Human	Gut microbiota of a SCZ patient after ECT	Stool sampling before and after ECT	After ECT
				↓ Scores in BPRS and BFCRS
				Gut microbiota differences before and after ECT
			Assessments (BPRS, BFCRS)	↓ *Clostridium*
				↑ *Lactobacillus*
			ECT (14 sessions)	
				↑ *Bacteroides*
He et al. [99]	Human	Gut microbiota differences	Stool sampling	↑ Clostridiales, Lactobacillales, and Bacteroidales in UHR vs HR and HC
			MRS scans	
				↑ SCFAs
			Recruitment (81 HR SCZ, 19 UHR SCZ, 69 HC)	↑ ACC choline in UHR vs HR and HC
		Choline concentrations in the ACC		
Yuan et al. [100]	Human	Metabolic parameters	Recruitment (41 SCZ, 41 HC)	↓ *Bifidobacterium*, *E. Coli*, and *Lactobacillus* in SCZ vs HC
			Assessment (PANSS)	↑ Clostridium
				Post 24-weeks risperidone
				↑ Body weight, BMI, hs-CRP, SOD, HOMA-IR
			Blood and stool sampling	
				↑ *Bifidobacterium* and *E. Coli*
				↓ *Clostridium* and *Lactobacillus*
				Only changes in *Bifidobacterium* correlated with changes in weight and BMI
			Standard enzymatic methods + electro-chemiluminescence immunoassay	
			Automatic biochemical analyzer	
			Particle-enhanced assay + chemical colorimetric assay	
			QIAamp fast DNA stool mini kit	
		SOD	qRT-PCR	
		hs-CRP		
		Gut microbiota		
		Metabolic/microbiota changes relationship		
		24-weeks risperidone treatment		
Schwarz et al. [101]	Human	Fecal microbiota	Assessment (extended BPRS, SANS, GAF, food habits, physical activity)	Similar α-diversity SCZ vs HC
				Difference in β-diversity
				↑ and ↓ different strains of Phyla (Proteobacteria, Fusobacteria, Firmicutes, Bacteroidetes)
			Stool sampling	In active SCZ patients:
				↑ Lactobacillaceae
				↓ Veillonellaceae
		SCZ-first episode, HC	qRT-PCR	
Okubo et al. [102]	Human	*B. breve* A-1 effects on SCZ-anxiety/depression, and the immune system	4-weeks *B. breve* A-1 admin	↑ HADS and PANSS scores after *B. Breve* A1
			Assessment (HADS, PANSS)	
				↑ Relative abundance of gut *Parabacteroides* after in *B. breve* A1
			Blood test findings	
			Fecal microbiota composition	
Flowers et al. [103]	Human	AAP treatment	Starch tolerability assessment	= overall microbiota composition at baseline between AAP vs non-AAP
			Stool sampling	
				↑ Alistipes in non-AAP
				AAP-microbiome varied with resistant starch admin
				↑ Actinobacteria phylum in AAP-treated
			PowerMag soil DNA isolation kit	
			16S rRNA gene MiSeq-based high throughput sequencing	
		6 months prebiotics (resistant starch)	Illumina expression array	
		Recruitment (37 SCZ, BPD)		
Ghaderi et al. [104]	Human	Effects of vitamin D/probiotic combination on metabolic and clinical SCZ symptoms	Assessment (PANSS, BPRS)	Vitamin D
			Vitamin D3/probiotic every 2 weeks or placebo (12 weeks)	↑ PANSS scores
				Vitamin D/probiotic co-supplementation
				↑ TAC
				↓ MDA, hs-CRP
				↓ Fasting plasma glucose, QUICKI, HOMA-IR
			Fasting blood sampling	
			ELISA	
			Antioxidant markers (TAC, GSH, MDA)	
			Insulin markers (HOMA-IR, QUICKI)	
			Enzymatic kits	
Zhu et al. [105]	Human, mice	FMT from SCZ humans to HC mice	Behavioral testing (OF, RSIT, TCST, NORT, FST, EPM, BM, TST)	SCZ-transplanted mice showed behavioral (cognitive and locomotor) impairments up to 10 days post FMT
		Cognitive and motor abilities in SCZ-induced mice	Mice stool sampling	
				↑ Kyn/KynA pathway of Tr degradation in both periphery and brain
			16S rRNA gene MiSeq-based high throughput sequencing	Increased DA in PFC, and 5-HT in HPC
			ELISA	
			qRT-PCR	
		Kyn/KynA pathway of Tr degradation		
		Extracellular DA in PFC, 5-HT in HPC		

The table provides a summary of the most relevant studies cited in the manuscript related to the role of the gut microbiota in the onset and development of SCZ

*PCP* phencyclidine; *SCZ *schizophrenia; *NORT* novel object recognition test; *HC* healthy controls; *GF* germ free; *GLU* glutamate; *OF* open field; *EPM* elevated plus maze; *DA* dopamine; 5-*HT* serotonin; *qRT*-*PCR* quantitative real time-PCR; *PFC* prefrontal cortex; *HPC* hippocampus; *Tr* tryptophan; *Kyn* kynurenine; *KynA* kynurenic acid; *hs*-*CRP* high-sensitivity C-reactive protein; *LBP* lipopolysaccharide-binding protein; *SCD14* soluble CD14; *GI* gastrointestinal; *G*/*R* genital/reproductive; *CFR* conditioning freezing response; *SI* social isolation; *RBANS* repeated battery for the assessment of neuropsychological status; *MDA* malondialdehyde; *SDS* schedule for deficit syndrome; *MINI* mini international neuropsychiatric interview; *PANSS* positive and negative syndrome scale; *BPRS* brief psychiatric rating scale; *GMEs* gut-microbiota associated epitopes; *MD* microbial dysbiosis; *GOGAT* glutamate synthase; *MWAS* metagenomic-wide association study; *ROC* receiver operating characteristic; *BPD* bipolar disorder; *ECT* electroconvulsive therapy; *BFCRS* Bush–Francis catatonia rating survey; *ACC* anterior cingulate cortex; *MRS* magnetic resonance spectroscopy; *HR* high risk; *UHR* ultra high risk; *SCFA* short-chain fatty acids; *SOD* antioxidant superoxide dismutase; *HOMA*-*IR* homeostasis model of assessment-insulin resistance; *SANS* assessment of negative symptoms; *GAF* global assessment of functions; *HADS* hospital anxiety and depression scale; *AAP* atypical antipsychotics; *TAC* total antioxidant capacity; *GSH* total glutathione; *QUICKI* quantitative insulin sensitivity check index; *RSIT* reciprocal social interaction test; *TCST* three-chamber social test; *BM* Barnes maze; *FST* forced swimming test; *TST* tail suspension test

However, it is still difficult to acknowledge a direct link between the gut microbiome and SCZ because a comprehensive animal model of SCZ covering all the broad spectra of SCZ symptoms is still missing. The available animal models of SCZ reflect four categories: developmental, drug-induced, lesion, or genetic manipulation [106]. Many rodent models have phenotypic behavioral changes similar to the “positive-like” symptoms of SCZ, reflecting altered mesolimbic dopamine function. The negative and cognitive impairments in SCZ are resistant to treatment with current antipsychotics, even after remission of the psychosis, limiting their therapeutic efficacy. Work has begun to identify specific rodent behavioral tasks with translational relevance to exact cognitive domains affected in SCZ [107], thus reporting the effect of current and potential antipsychotics on these tasks.

Nevertheless, the scientific community needs to develop more comprehensive animal models that more adequately replicate deficits in each aspect of SCZ. Increasing information on the neurochemical and structural CNS changes accompanying each model will also help assess treatments that prevent the development of SCZ rather than treating the symptoms. Understanding the SCZ etiology is another pivotal change required to enable new, more effective therapeutic strategies to be developed.

In the same framework as the development of model studies, it has been reported that the GI microbiota contributes to the pathophysiology of brain-derived neurotrophic factor (BDNF) in selected brain regions and the related neurotransmission, including altered NMDAR expression [108]. Another study hypothesized that modulation of BDNF levels affects NMDAR function, possibly leading to psychiatric disorders [109]. Finally, a connection between SCZ patients, an altered gut immune system, and metabolic syndromes have been described [110]. The GI microbiota-BDNF-NMDAR interactions indicate the importance of normal gut microbiota in NMDA-dependent hippocampal memory. We believe that this leads to the idea that it may be possible to treat SCZ cognitive dysfunctions preventatively through the manipulations of bacterial populations.

It has been found that the use of prebiotic diets may increase the cortical neuronal response to NMDARs and therefore improve cognitive flexibility [111]. Another study showed that certain strains of probiotics (*Lactobacillus Rhamnosus*) might increase and decrease the expression of GABAa and GABAb receptors in different brain areas, inducing anxiety and depressive-like behavioral responses [25]. Taken together, these pieces of evidence imply that an unbalanced gut-brain NMDA/GABA interaction may affect those affective/cognitive conditions, resembling the ones from SCZ. Another study investigated the effects of the gut microbiota in a rat model of SCZ induced by subchronic PCP administration [22]. The model works by injecting rodents with PCP systemically twice a day, for at least a week, to have a chronic manifestation of the SCZ features. Afterward, a washout period of seven days is needed, after which the subjects exhibit SCZ-like behaviors, such as hyperlocomotion, learning and memory impairments, and disrupted executive functions. The authors found that PCP-induced SCZ-like symptoms are at least partly mediated by the gut microbiota, as its removal by antibiotics abolishes the effect of PCP. We think this is consistent with the idea that specific SCZ symptoms may result from gut microbial dysbiosis and receptor malfunction. It is worth noting that sets of antibiotics (used to make germ free animals) induce gut dysbiosis through a direct pharmacological effect on the CNS via the vagus nerve, particularly the modification of GABA-mediated neurotransmission, and the actions of benzodiazepines [23–25]. Thus, it is possible to argue: (1) the importance of normal gut microbiota in linking NMDAR-GABA activity with HPC memory, motor control, and cognitive flexibility; and (2) that the altered gut microbiota can mimic at least in part the selective pharmacological active antagonism to NMDARs of MK-801 widely used to create a pharmacological model of SCZ. Our idea is that this impairment in cognitive function, associated with a reduction in NMDAR-GABA levels, may be preventable or treatable by manipulating the gut bacterial populations or through a direct restoration of NMDAR-GABA activity.

Commensal organisms (*Lactobacillus* and *Bifidobacterium* strains) in the gut can act on central GABAa, and GABAb receptors via the vagus nerve and are associated with reduced stress and anxiety response [25]. Investigators have reported that several bacteria produce GABA [112–114]. Moreover, active manipulation of the gut microbiota induces GABA activity in the CNS, restoring such activity lost due to refractory epilepsy [115] and obesity [116]. The crucial role of GABA signaling regulates vagal neuronal activity, and this accompanies gastric motility traces. However, how GABA produced by the microbiota may be involved in SCZ remains to be elucidated. In our view, more investigations on this topic are crucial. The gut microbiota and SCZ-like cognitive dysfunctions are both arousing the interest of neuroscientists because both of these actors have a crucial role in the predictivity of SCZ. In our view, both the gut microbiota and SCZ-like cognitive dysfunctions can be considered as predictive endophenotypes of SCZ as a “wide-spectrum” psychiatric disease.

## Lines of intervention

### Probiotic dietary intake (psychobiotics) and fecal transplant

Recently, investigations of potential therapies have aimed at normalizing altered microbiota signaling to the ENS and the CNS. One of these takes advantage of probiotic diets acting on mood disorders (depression, anxiety; [25]) and cognitive functions (learning and memory; [117]). Probiotics are currently defined as a live organism that exerts a health benefit when ingested in acceptable amounts [118]. Some *Lactobacillus* and *Bifidobacterium* strains secrete GABA, regulating several psychophysiological processes [119]. This finding led to the idea that probiotics may functionally act as vehicles for neuroactive drugs, thus acting as potential psychotropic agents [120]. Therefore, Dinan and Cryan defined a “psychobiotic” as a live organism that produces a health benefit in patients suffering from a psychiatric illness [121]. This probiotics class can produce and deliver many neurotransmitters such as acetylcholine and serotonin via the vagal afferents. In one experiment, animals fed with *Lactobacillus Rhamnosus* showed reduced anxiety on various behavioral measures, with an altered central expression of the GABAa and GABAb receptors. To determine the mechanism of action, the animals underwent vagotomy or sham surgery. Vagotomy prevented the emergence of an anxiolytic effect from the probiotic and prevented changes in GABA receptor expression [25].

Furthermore, in another study, probiotic supplementation was delivered to a diabetes rat model that performed the Morris Water Maze, with excitatory postsynaptic potentials (EPSPs) recorded from CA1 hippocampal regions. Results showed that the probiotic mixture caused improved learning and memory in this model [117]. In our view, these studies gave only a hint of what may be possible to achieve in the neurophysiological research of psychiatric illnesses such as SCZ by exploiting the link between probiotic dietaries and GABA central activity. The “psychobiotic” concept brought up by Dinan and Cryan reveals fertile ground in which it is possible to plant a new seed in the main field of neuroscience research, connecting the classic field of the neurophysiology of psychiatric disorders and nutrigenomics.

Significant potential in recent years comes from the use of a new technique that allows a transfer of fecal bacteria and other healthy microbes from one species to another. As was mentioned above, FMT is an experimental/therapeutic approach that alters the gut flora drastically. In contrast to probiotics that contain only a few bacterial species, FMT contains thousands of species native to the human gut. Many studies have shown the efficacy of FMT for treating diseases such as irritable bowel syndrome (IBS) and insulin sensitivity [116, 122]. Moreover, FMT may also help alleviate some psychiatric disorders [123–125].

Conversely, it has been shown that FMT from SCZ subjects to specific pathogen-free mice induced SCZ-like alterations as the learning and memory impairment and hyperlocomotion in those mice [105]. Maternal separation and perinatal stress animal models are potent tools for studying neurodevelopmental factors in the pathogenesis of SCZ [126]. For instance, adult rats with maternal separation showed altered fecal microbial composition compared with usually reared control animals [127].

Altogether, the importance of both psychobiotics and FMT has only recently been acknowledged. Although only a few preclinical and clinical studies have been carried out in this field, these findings allow us to strongly consider the gut microbiota as a potential target for treating neuropsychiatric disorders, representing an effective therapeutic option. Moreover, implementing these therapies can be incredibly beneficial regarding side effects, cost, and ease of implementation compared to previous pharmacological therapies.

### Optogenetic manipulation in the brain and gut

In the last 2 decades, in vivo optogenetic methods have recognized brain areas and circuits guiding certain behaviors in freely moving rodents by using a combination of light and viral vectors [128].

Through the expression of engineered rhodopsins, virally vectored neurons can be activated or inhibited by a specific light wavelength with channelrhodopsin (ChR2) or halorhodopsin (NpHR) respectively [129]. This method allows us to investigate the function of brain circuits from the perspective of both excitation and inhibition. Specifically targeting terminals with microbial opsins transfected locally into the brain of transgenic rodents will alter specific neurotransmitters released through their downstream nuclei. Real-time behavioral changes make optogenetics a powerful method that can reveal which specific neuronal ensemble might modulate specific psychiatric misbehaviors, such as major depressive syndrome, anxiety-like behaviors, or SCZ [130]. In this regard, recent studies applied optogenetics to study the role of the hypofunctioning NMDAR onto PV  +  interneurons in the mainframe of cognitive impairments associated with SCZ. Contemporary photoactivation of PNs with ChR2 and photoinhibition of PV  +  with NpHR induced altered gamma oscillations, respectively, is a hallmark of learning and memory impairment in SCZ [131]. Moreover, Carlen et al. found out that mice lacking NMDAR neurotransmission only in PV  +  interneurons display enhanced cortical gamma oscillations and that optogenetic activation of PV  +  on those mice impaired the synchronization of gamma oscillations, therefore inducing impairments in working memory and associative learning, which are characteristic of cognitive impairments in SCZ [79]. More recently, another study reproduced a PFC PV  +  activity reduction on mice using optogenetic inhibition, aiming to evaluate the hypothesis that a reduced cortical PV  +  may increase an excitatory/inhibitory imbalance, which has been considered another critical feature of SCZ-like cognitive dysfunctions [132].

On the other hand, optogenetic activation or inhibition of PNs in the HPC induced or reduced the behavioral effects of SCZ, respectively [133, 134]. Wolff and colleagues induced optogenetic over-activation of ventral HPC PNs based on the hypotheses that the excitatory over-activity of the HPC-CA1 region in humans is a potential predictive marker of SCZ-like psychosis. Results showed that optogenetic Chronos activator in mice ventral HPC increased abnormally PNs activity inducing hyperlocomotion, a SCZ-positive symptoms hallmark, and impaired performance on the spatial novelty preference, that is an SCZ-cognitive impairment. Conversely, the recent study carried by Fan et al. [134] applied a protocol where prior administration of PCP induced significant impairment in the acquisition of long stimulus-induced delay eye blink conditioning. Following, optogenetic inhibition of PNs of bilateral ventral HPC neurons alleviated the decreased acquisition and impaired conditioning, this driving the idea that increased activity in the HPC network plays a pivotal role in SCZ.

Very recently, studies have shown the ability of either ChR2-photoactivation or NpHR-photoinhibition to act on fibers originating from limbic structures and having an effect on gut colonic sensitivity in freely moving rodents [135]. Optogenetic applications with behavioral assessments of visceral pain were combined to address this goal. Firstly, AAVs containing ChR2 or NpHR were transfected at the amygdala’s central nucleus (CeA), and optic fibers were implanted at the bed nucleus of the stria terminalis (BNST). After a few weeks for recovery and ChR2/NpHR expression, isobaric colonic distension was measured with and without light applications. The researchers found that ChR2-photoactivation induced colonic hypersensitivity, while NpHR-photoinhibition did not have any effect. This approach shows that optogenetic activation of limbic brain nuclei can be used to advance our understanding of the complex visceral nociceptive circuitry in freely moving rodents.

In another study, optogenetic stimulation with ChR2 of GABAergic transporters (VGATs) of the zona incerta (ZI), which is encapsulated in between the subthalamic nuclei, induced maladaptive binge-eating in mice [136]. This study suggested an unexpectedly robust orexigenic potential for ZI GABAergic neurons. Specifically, the question was whether the paraventricular thalamic nucleus (PTN)-ZI GABAergic connections were crucial for maladaptive food intake regulation. In vivo stimulation of axon terminals from ZI GABA neurons to PTN glutamate neurons evoked food intake behavior, while 10 min continuous stimulation increased the intake of high-fat, sweet, and regular foods. Further, the authors demonstrated that the ZI GABA neurons’ optogenetic stimulation generated a more robust feeding response than the much-studied lateral hypothalamus stimulation, suggesting that the ZI GABA neurons can play a substantive role in enhancing food consumption.

Moreover, focal applications of light have been used to control ENS excitability to evoke propagating contractions, thus increasing colonic transit in rodent models of binge eating [137]. Hibberd and colleagues isolated colons from transgenic mice with Cre-mediated expression of ChR2 in calretinin neurons and analyzed them by immunohistochemistry, patch-clamp, and calcium imaging methods. Meanwhile, colonic motility was assessed using mechanical, electrophysiological, and video recording in vitro and fecal output in vivo. When focal light stimulation was applied to calretinin enteric neurons, polarized motor reflexes were evoked, followed by premature anterograde propagating contractions. Thus, light stimulation could evoke motility from sites along the entire colon. Interestingly, the researchers implemented a new “wireless light-emitting diode” implanted onto the colon wall. The approx. 10 mm diameter device includes a conductive receiver coil, a capacitor, and a rectifier. A ductile connecting trace includes metal lines for power transmission from the coil to a μLED on an injectable needle, with an illumination area of about 0.06 mm^2^. Power is transferred to the coil by magnetic coupling to a transmission antenna and then emitted via electromagnetic waves at a particular radiofrequency. The intragastric surgery to implant the device uses an insertion between the skin and peritoneum. The needle containing the μLED is inserted through an incision in the peritoneum. Finally, the μLED is placed next to the proximal colon, just distal to the cecal-colonic junction. This technical improvement allowed the researchers to verify in vivo the results obtained in vitro. In fact, by applying focal light via the wireless device onto the colon, Hibberd et al. significantly demonstrated that freely moving mice enhanced the number of fecal pellets, confirming the previous in vitro results. One primary outcome of this study is the technical advancement of a new tool that might be key in studying how optogenetic control may directly affect gut motility.

It is important to note that optogenetics utilizes light-sensitive channel pumps (ChR2s) or light-driven pumps (NpHRs), and as such, are so-called “actuators” having properties with depolarizing or hyperpolarizing cells in response to specific light waves [138]. The next step will be to consider targeting specific actuators to desired cells or regions in the ENS. Viral vectors and transgenic animals are the most widely used tools to achieve this purpose in the CNS. A specific promoter or local injection could achieve high spatial resolution (i.e., specific cell type or restricted region). Therefore, several viral vectors, including retroviruses and adenoviruses, and adeno-associated viruses (AAV), have been used for delivery to the GI tract. A few AAV serotypes seem to be efficient [139–141]. Indeed, it is possible to control ENS neuron activities optogenetically, thus providing a new strategy for treating ENS neuronal diseases. For example, although the details of the molecular mechanisms are still not completely known, it might be possible to selectively activate the gut-brain dopaminergic pathway to investigate the molecular mechanisms of Parkinson’s disease (PD) and aberrant motivations leading to addiction and binge eating. Moreover, it would be exciting to test whether light activating dopaminergic enteric neurons, which express optogenetic actuators, could rescue dopaminergic degeneration and locomotor dysfunction behavior. To this aim, one could target optogenetic actuators to dopaminergic neurons in the ENS of animal models of the diseases mentioned above.

In our view, optogenetics opens up the exciting possibility of manipulating the ENS, though challenges for such an application remain. Much work is needed to identify new specific promoters in the ENS that remain unknown, compared to the CNS where various promoters are well known and available. An additional research challenge is to create more significant promoters, considering the small size of the virus’s packaging capacity. Another valuable model for studying the ENS is the zebrafish [142–144], which has unique advantages for the optogenetic approach. In particular, there are many transgenic lines, and they are transparent in early life stages, which means easy optical access and simplifying light stimulating equipment.

### Conclusions and remarks

In this review, we addressed several questions regarding the relationship between gut microbiota and SCZ. Among the most recent findings concerning SCZ, among the most interesting is the “NMDA glutamatergic receptor hypofunctioning hypotheses”, formulated based on evidence that NMDAR antagonists are involved in positive, negative, and cognitive impairments of SCZ in healthy subjects [7, 58]. Recent studies have also revealed a significant role for GABAergic transmission as an SCZ endophenotype [10–12, 68]. As a matter of fact, in vivo research indicates that extracellular GABA is lower in subjects with SCZ, particularly in PV  +  interneurons [59, 76, 77]. Finally, in vivo optogenetics has demonstrated the crucial role of PV  +  interneurons on GABAergic inhibitory activity, overcoming NMDAR hypofunction induced by NMDAR antagonists [74, 79]. However, although recent findings have revealed the specific role of inhibitory transmission in SCZ symptomatology, knowledge is still missing regarding the brain areas and their circuitry possibly involved in this pathology. Very recently, it has been hypothesized that a PFC-hippocampus circuit may have a role in the etiology of SCZ cognitive inabilities [145–148].

Nevertheless, we have seen that it is questionable to consider SCZ to be a condition caused by the aberrant function of a single neurotransmitter, even in the hypothesized PFC-hippocampal circuit. From our point of view, investigating only one line of neurotransmission is likely an outdated approach. Researchers may use more complex experimental designs with modern technological methods, where at least two actors are investigated. It has become increasingly evident that psychiatric diseases such as SCZ have a complicated “wide-spectrum” symptomatology, reflecting significant complexity in the brain structures affected by these pathologies, with different actors playing simultaneously. In vivo optogenetics may help investigate the exact role of individual neurotransmitters in the PFC-hippocampal circuit to elucidate the origins of SCZ-cognitive inabilities.

We also reviewed recent studies related to the gut microbiota and their growing relationships with a constellation of psychopathologies, ranging from the neurodegenerative (PD, AD), psychiatric (SCZ and autism), to psychological (depression, anxiety, addiction, eating disorders). Significantly, recent studies have hypothesized a connection between SCZ and a decreased microbiome α-diversity index compared with healthy controls [85]. Further, commensal organisms (*Lactobacillus* and *Bifidobacterium* strains) can produce GABA in the gut via the vagus nerve, accompanying changes in cerebral GABAergic activity [25]. However, how GABA produced by the microbiota may be involved in SCZ remains to be elucidated. Indeed, much research has been focused on more psychological diseases, such as anxiety and depression. Psychiatric disorders such as SCZ are still relatively neglected from projects and studies. We believe that this might be because explaining the role of excitatory brain transmission in gut dysbiosis and how the CNS-ENS system interacts more directly and more straightforward to depict than inhibitory transmissions, which imply, for instance, feedback and feedforward activations. However, due to recent findings implicating the enormous role of gut dysbiosis in NMDAR-GABA transmission leading to psychiatric disturbances, we strongly think it is essential to pursue investigations on this path.

We also summarized some new therapeutic possibilities to treat psychiatric illnesses by acting on the gut microbiota. Probiotic diets, fecal transplants, and especially optogenetic manipulations of both the CNS and ENS might be effective techniques that deserve implementation. It would be fascinating to study the gut microbiota-SCZ relationship in detail using in vivo optogenetics, manipulating either the brain or the gut actors. To our knowledge, no study to date has systematically explored the hypotheses of an optogenetic gut manipulation driving reduced GABAergic activity in those brain areas related to SCZ-like cognitive functioning. This kind of study might improve research on potential treatments, and it will undoubtedly increase our knowledge on how the gut microbiota can manipulate GABA production and therefore lead to changes in CNS GABAergic activity.

Nevertheless, pursuing such a research path might be very difficult both at theoretical and technical levels. For example, at the technical level, it is necessary to develop new micro optogenetic tools that are implantable in the gut. The wireless µLED of Hibberd et al. [137] is an example of the type of breakthrough needed to boost research. Connecting the gut and brain using more sophisticated wireless devices would be an enormous step in this new field.

At the theoretical level, investigations on GABAergic transmission related to psychiatric disorders are rapidly multiplying, but developing studies involving the CNS-ENS, gut dysbiosis, and GABA inhibitory transmission could be excessively complex at this point. In our view, several more intermediate steps have to be done to deeply understand the role of GABA activity in psychiatric disorders. For example, it is likely necessary to investigate GABA along with every single other actor (CNS excitatory neurotransmission, ENS acetylcholinergic transmission, probiotics), via the vagus nerve (vagotomy), through the brain (areas where GABA is well known to be involved in psychiatric diseases). Once this mission is accomplished and more technical issues will be solved, there will still be time and space to develop new and more solid studies on the role of GABA inhibitory transmission in the gut microbiota in the context of psychiatric disorders such as SCZ.

## Data Availability

Not applicable.

## Abbreviations

AAV: Adeno-associated virus
AD: Alzheimer’s disease
BDNF: Brain-derived neurotrophic factor
BNST: Bed nucleus of the stria terminalis
CA1: Cornus ammonis 1
CeA: Amygdala’s central nucleus
ChR2: Channelrhodopsin2
CNS: Central nervous system
DIO: Double-floxed inverted open reading frame
DSM-5: Diagnostic and Statistic Manual of Mental Disorders, 5th edition
ENS: Enteric nervous system
EPSP: Excitatory postsynaptic potential
FMT: Fecal microbiota transplantation
GABA: Gamma-aminobutyric acid
GI: Gastrointestinal
GluN1: Glutamate ionotropic receptor subunit 1
GluN2: Glutamate ionotropic receptor subunit 2
HPA: Hormonal-pituitary-adrenal
HPC: Hippocampus
HR3.0: Halorhodopsin
IBS: Irritable bowel syndrome
IEG: Immediate-early-gene
MEG: Magnetoencephalography
MIA: Maternal immune activation
MK-801: 5-Methyl-10,11-dihydro-5H-dibenzo[a,d]cyclohepten-5,10-imine
NES: Neuroendocrine system
NMDAR: N-methyl-D-aspartate receptor
PCP: Phencyclidine
PD: Parkinson’s disease
PFC: Prefrontal cortex
PING: Pyramidal interneuron network gamma
PNNs: Perineuronal nets
PNs: Pyramidal neurons
Poly I:C: Polyinosinic–polycytidylic acid
PTN: Paraventricular thalamic nucleus
PV+: Parvalbumin positive interneurons
SCZ: Schizophrenia
VGATs: GABAergic transporters
ZI: Zona incerta
